# Genetic overlap between idiopathic pulmonary fibrosis and COVID-19

**DOI:** 10.1183/13993003.03132-2021

**Published:** 2022-07-07

**Authors:** Richard J. Allen, Beatriz Guillen-Guio, Emma Croot, Luke M. Kraven, Samuel Moss, Iain Stewart, R. Gisli Jenkins, Louise V. Wain

**Affiliations:** 1 Department of Health Sciences, University of Leicester, Leicester, UK; 2 Department of Genetics and Genome Biology, University of Leicester, Leicester, UK; 3 National Heart and Lung Institute, Imperial College London, London, UK; 4 National Institute for Health Research, Leicester Respiratory Biomedical Research Centre, Glenfield Hospital, Leicester, UK; 5 These authors contributed equally

## Abstract

Coronavirus disease 2019 (COVID-19) is an infectious disease potentially leading to long-lasting respiratory symptoms and has resulted in over 4 million deaths worldwide. Idiopathic pulmonary fibrosis (IPF) is a chronic interstitial lung disease (ILD) characterised by an aberrant response to alveolar injury leading to progressive scarring of the lungs. Individuals with ILD are at a higher risk of death from COVID-19 [1].

## Abstract 2

**Positive genetic correlations for COVID-19 and IPF point to interferon-mediated innate immunity in both response to infection and chronic disease whilst negatively correlated signals highlight implications for drug targeting** https://bit.ly/37MMxZa

*To the Editor*:

Coronavirus disease 2019 (COVID-19) is an infectious disease potentially leading to long-lasting respiratory symptoms and has resulted in over 4 million deaths worldwide. Idiopathic pulmonary fibrosis (IPF) is a chronic interstitial lung disease (ILD) characterised by an aberrant response to alveolar injury leading to progressive scarring of the lungs. Individuals with ILD are at a higher risk of death from COVID-19 [1].

Large genome-wide association studies (GWAS) have identified multiple genetic signals associated with severe COVID-19 [2], including a signal within the *DPP9* gene that is also associated with increased IPF risk [3]. GWAS have identified 20 genome-wide significant signals of association with IPF risk [4, 5] with the largest genetic risk factor being a common variant located in the promoter region of *MUC5B* (rs35705950, odds ratio >4). Previous analyses suggest IPF is a causal risk factor for severe COVID-19 but noted that the effect of rs35705950 was in the opposite direction (*i.e.* the allele associated with increased risk of IPF was protective for severe COVID-19) [6].

We aimed to further explore the shared genetic architecture and identify novel shared genetic loci between the two diseases, using new enlarged GWAS of IPF and COVID-19 risk.

We used the largest GWAS of IPF risk, which consisted of unrelated European individuals from across five studies [5]. Cases were selected from centres in the USA, UK and Spain, diagnosed using American Thoracic Society and European Respiratory Society guidelines. This data is available to access from https://github.com/genomicsITER/PFgenetics.

For COVID-19, the summary statistics from version 6 of the COVID-19 Host Genetics Initiative (HGI_v6, available to access from www.COVID19hg.org/results/r6/) were used. This analysis considered four different COVID-19 phenotypes according to the severity of the disease and the controls used: A2: very severe respiratory confirmed COVID-19 *versus* population; B1: hospitalised COVID-19 *versus* not hospitalised COVID-19; B2: hospitalised COVID-19 *versus* population; and C2: COVID-19 *versus* population. The COVID-19 phenotypes A2, B1 and B2 capture both susceptibility and severity of COVID-19, while phenotype C2 captures only susceptibility to COVID-19 infection.

Using LD Score Regression [7], we calculated the genome-wide genetic correlation between IPF and the four COVID-19 phenotypes. There was a significant weak positive genome-wide correlation between IPF and COVID-19 severity phenotypes (A2: r^2^=0.274, p=0.0045; B1: r^2^=0.279, p=0.0093; and B2: r^2^=0.261, p=0.0005) but not with COVID-19 infection (C2: r^2^=0.066, p=0.433).

We investigated the 20 previously reported IPF genetic association signals [4, 5] for their association in the four COVID-19 GWAS, and 26 variants reaching genome-wide significance in the COVID-19 GWAS were tested for their association with IPF (proxy variants, r^2^>0.8 in European population, were investigated if the top associated variant was not included). At genetic loci showing an association with both traits (after Bonferroni correction for multiple testing), we investigated whether the same causal variant was driving both the IPF and COVID-19 associations using coloc [8]. Regions with a posterior probability >80% of there being a shared causal variant (assuming up to one causal variant for each trait in the region and that variant has been measured) were deemed to have colocalised. Four genetic association signals showed evidence of a shared causal variant between IPF and at least one COVID-19 phenotype (posterior probability >80%), namely loci at 7q22.1, near *MUC5B*, near *ATP11A* and near *DPP9* (table 1). The 7q22.1 locus has not previously been reported for association with COVID-19. Three additional IPF genetic signals (at 17q21.31, *DSP* and *DEPTOR*) showed an association with COVID-19 but did not colocalise, suggesting there are different causal variants between the two traits at these loci. Visual inspection of the 17q21.31 locus revealed extended linkage disequilibrium (due to the presence of a large inversion) meaning colocalisation analyses could not determine whether there were shared or distinct causal variants.

**TABLE 1:** Variants reaching Bonferroni-corrected significance for both idiopathic pulmonary fibrosis (IPF) and coronavirus disease 2019 (COVID-19)

**chr:position** **rsid**	**REF/EFF**	**IPF**	**COVID-19 phenotypes**				**Gene expression** **(tissue, coloc)**	**PheWAS**
			**A2**	**B1**	**B2**	**C2**		
		**OR (95% CI)** **p-value**	**OR (95% CI)** **p-value** **coloc**	**OR (95% CI)** **p-value** **coloc**	**OR (95% CI)** **p-value** **coloc**	**OR (95% CI)** **p-value** **coloc**		
**chr7:99630342** **rs2897075**	C/T	1.30 (1.23, 1.37) p=1.77×10^−21^	1.07 (1.04, 1.12) p=1.63×10^−4^ coloc=88.2%	1.02 (0.99, 1.05) p=0.238	1.04 (1.02, 1.06) p=5.55×10^−4^ coloc=48.1%	1.01 (1.00, 1.02) p=0.014	Decreased *ZKSCAN1* (blood, 99.4%) Decreased *TRIM4* (blood, 85.4%)	• Lung function (FEV_1_/FVC, PEF) • Chronic obstructive pulmonary disease • Blood traits (mean corpuscular haemoglobin and volume, red cell distribution width, red blood cell count, mean corpuscular volume, mean corpuscular haemoglobin concentration, platelet count, mean platelet volume) • Impedance of leg right • Low density lipoprotein cholesterol levels
**chr11:1241221** **rs35705950**	G/T	5.06 (4.67, 5.47) p=9.09×10^−418^	0.83 (0.77, 0.89) p=1.17×10^−7^ coloc=100%	0.89 (0.84, 0.94) p=2.20×10^−5^ coloc=98.5%	0.89 (0.86, 0.93) p=1.22×10^−8^ coloc=100%	0.99 (0.98, 1.01) p=0.448	Increased *MUC5B* (lung, 100%)	-
**chr13:113534984** **rs9577395**	G/C	1.29 (1.21, 1.38) p=4.78×10^−14^	0.90 (0.87, 0.94) p=4.38×10^−6^ coloc=99.0%	0.94 (0.90, 0.97) p=8.76×10^−4^ coloc=52.1%	0.94 (0.91, 0.96) p=8.67×10^−7^ coloc=99.5%	0.99 (0.98, 1.00) p=0.037	Increased *ATP11A* (blood, 99.6%)	• Blood traits (mean corpuscular volume, mean corpuscular haemoglobin, red cell distribution width, platelet count, red blood cell count) • HbA1c • Lung function (FEV_1_/FVC)
**chr19:4717672** **rs12610495**	A/G	1.28 (1.21, 1.36) p=2.56×10^−16^	1.20 (1.15, 1.26) p=1.64×10^−15^ coloc=97.9%	1.08 (1.04, 1.11) p=1.73×10^−5^ coloc=98.5%	1.11 (1.09, 1.14) p=6.09×10^−18^ coloc=97.9%	1.03 (1.02, 1.04) p=5.10×10^−10^ coloc=98.1%	Decreased *DPP9* (blood, 88.3%)	• Appendicular lean mass

chr: chromosome; REF: reference allele; EFF: effect allele (*i.e.* the variant the effect estimates are in relation to); FEV_1_: forced expiratory volume in 1 s; FVC: forced vital capacity; PEF: peak expiratory flow; HbA1c: haemoglobin type A1c; PheWAS: phenome-wide association study; ILD: interstitial lung disease. COVID-19 phenotypes are as follows. A2: very severe respiratory-confirmed COVID-19 (8779 cases) *versus* population (1 001 875 controls); B1: hospitalised COVID-19 (14 408 cases) *versus* not hospitalised COVID-19 (73 191 controls); B2: hospitalised COVID-19 (24 274 cases) *versus* population (2 061 529 controls); C2: COVID-19 (112 612 cases) *versus* population (2 474 079 controls). The coloc values give the posterior probability there is a shared causal variant between IPF and that COVID-19 phenotype at that genetic loci. Colocalisation analyses were only performed on signals showing a possible association with both traits after correcting for multiple testing. Percentages shown in the gene expression column are the posterior probability of colocalisation between the IPF risk signal and the gene expression eQTL signal in the tissue stated (only genes with posterior probability >80% are presented in the table). For the PheWAS results, phenotypes where the variant had p<10^−5^ and which colocalised with the IPF signal (posterior probability >80%) are presented. Only non-ILD and non-COVID-19 phenotypes were investigated in PhenoScanner, Open Targets and GWAS Catalog for the PheWAS analysis. Proxy variants (with r^2^>0.8) were also investigated in PhenoScanner. For Open Targets only traits with genome-wide summary statistics from GWAS Catalog were investigated.

For the four genetic loci shared between IPF and at least one COVID-19 phenotype, we investigated whether shared genetic signals were associated with gene expression in lung tissue (GTEx_lung [9], n=515) and whole blood (eQTLGen [10], n=31 684). Where the variant met a false discovery rate of 5%, colocalisation analyses were performed using coloc and deemed to be linked to gene expression if the posterior probability of a shared causal variant was greater than 80%. Three of the four shared signals colocalised with expression of the single nearest gene in blood or lung (*MUC5B*, *ATP11A* and *DPP9*) (table 1). The IPF and COVID-19 risk increasing alleles at the 7q22.1 signal colocalised with decreased expression of *ZKSCAN1* and *TRIM4* in blood.

Finally, we performed a phenome-wide association study (PheWAS) to identify if the overlapping genetic signals had been previously reported for association with other traits (p<10^−5^) using publicly available resources (PhenoScanner_v2, GWAS Catalog and Open Targets). Colocalisation analyses were performed to determine if the same causal variant was driving both traits. The signal on chromosome 7 was associated with a number of blood traits and the signal near *ATP11A* was associated with blood traits and HbA1c (average blood glucose levels, used in diagnosing diabetes) (table 1). The IPF and HbA1c signals did not colocalise; however, as diabetes is a risk factor for COVID-19 [11], we further investigated the effects on gene expression for this signal in all GTEx tissues. The allele (rs423117_T, the sentinel variant from the Hb1AC GWAS) associated with higher Hb1AC levels was associated with increased *ATP11A* expression in liver and decreased expression in cultured fibroblasts, but there was no association with *ATP11A* expression in blood.

In summary, genetic association signals near *MUC5B*, *DPP9* and *ATP11A* have previously been reported for both COVID-19 severity and IPF risk; we show for the first time that these signals are likely due to the same underlying causal variant. In addition, we show the signal at 7q22.1 associated with IPF also shows a novel association with COVID-19 and implicates *TRIM4* and *ZKSCAN1*.

Despite a positive genome-wide genetic correlation between IPF risk and the COVID-19 severity phenotypes (A2, B1 and B2), we show that two of the four shared signals (at *MUC5B* and *ATP11A*) have opposite directions of effect on risk for the two diseases. The allele associated with increased risk of IPF and increased *ATP11A* expression in blood (rs9577395_C) was associated with decreased risk of severe COVID-19. The lipid flippase ATP11A has been suggested to have an important role in the innate immune response, and a depletion of this protein in human cells has been related to an increased inflammatory response [12]. Therefore, an increased expression of *ATP11A* may lead to better COVID-19 outcomes by attenuating chronic inflammation following initial infection. Our PheWAS highlighted a potential link with HbA1c and diabetes risk at this locus *via ATP11A* expression, although effects were tissue dependent. The IPF risk allele at *MUC5B* may have a protective effect in airway defence in patients with COVID-19 [6]. These findings of opposite genetic and tissue effects potentially highlight important differences between development of long-term chronic disease and response to infection, which could have implications when considering new drug targets.

The rs2897075_T allele at 7q22.1, associated with increased IPF and COVID-19 risk, was linked to decreased *TRIM4* and *ZKSCAN1* expression. TRIM4 is an important regulator of virus-induced interferon induction pathways and a proteomic study identified significant adjacency between SARS-CoV-2 M protein and TRIM4 [13]. Viral infection-induced micro-injury to the alveolar epithelium is thought to be a trigger for development of IPF [14], suggesting the interferon-mediated innate immune response could be central to both risk of chronic lung disease and worse outcomes due to SARS-CoV-2 infection. We also showed that the IPF and COVID-19-risk variant at *DPP9* was associated with a reduced *DPP9* expression. This serine dipeptidyl peptidase inhibits inflammasome activation [15] and has been related to antigen presentation [16], having an important role in the immune response. Further functional studies are required to better understand the specific role of these genes in the development of IPF and in response to COVID-19 infection.

Loci previously implicated by IPF GWAS relating to telomere dysfunction (*TERT*, *TERC*, *RTEL1*) and mitotic spindle assembly (*KIF15*, *MAD1L1, SPDL1*, *KNL1*) were not associated with COVID-19.

The colocalisation analyses assume a single measured causal variant. Although conditional analyses found no evidence of multiple independent association signals at the regions studied, we cannot guarantee all causal variants were measured. Furthermore, we utilised whole blood and lung tissue for gene expression so we cannot rule out cell-specific effects. A limitation of our analysis are the population groups used. Given the difficulties in selecting controls for infection GWAS [17], we used all of the HGI COVID-19 GWAS, which used four different COVID-19 phenotypes. We found that the genetic correlation results were almost identical across the three COVID-19 severity phenotypes (A2, B1 and B2). This suggests that variation in the colocalisation results may be due to variation in power as a consequence of different sample size and chance of misclassification in the COVID-19 GWAS. Secondly, to maximise the power of the analysis we utilised the largest GWAS of IPF and COVID-19 available. The IPF GWAS included only European individuals; however, the COVID-19 GWAS was performed as a multi-ancestry analysis with the majority of individuals being from European populations. Further analyses in non-European populations could help identify other overlapping ancestry-specific effects.

In conclusion, using the largest IPF and COVID-19 GWAS to date, we show there is a positive genome-wide genetic correlation between IPF and severe COVID-19 risk. However, some IPF-related pathways may have an opposite (*e.g. MUC5B* and *ATP11A* pathways) effect on severe COVID-19 risk.

## Shareable PDF

10.1183/13993003.03132-2021.Shareable1 This one-page PDF can be shared freely online.
Shareable PDF ERJ-03132-2021.Shareable
