# Supplementary material for: Genetic overlap between idiopathic pulmonary fibrosis and COVID-19
Source: Eur Respir J. 2022 Jul 7;60(1):2103132. doi: 10.1183/13993003.03132-2021 (PMC9130756; doi:10.1183/13993003.03132-2021)
Supplement: Supplementary file 1 [file ERJ-03132-2021.Shareable.pdf]

SHAREABLE PDF

Genetic overlap between idiopathic pulmonary fibrosis and COVID-19

Richard J. Allen[1,5], Beatriz Guillen-Guio[1,5], Emma Croot[2], Luke M. Kraven[1], Samuel Moss[3], Iain Stewart[3], R. Gisli Jenkins[3] and Louise V. Wain[1,4]

[1]Department of Health Sciences, University of Leicester, Leicester, UK. [2]Department of Genetics and Genome Biology, University of Leicester, Leicester, UK. [3]National Heart and Lung Institute, Imperial College London, London, UK. [4]National Institute for Health Research, Leicester Respiratory Biomedical Research Centre, Glenfield Hospital, Leicester, UK. [5]These authors contributed equally.

Corresponding author: Richard J. Allen (rja34@leicester.ac.uk)

Check for updates

Shareable abstract (@ERSpublications)
Positive genetic correlations for COVID-19 and IPF point to interferon-mediated innate immunity in both response to infection and chronic disease whilst negatively correlated signals highlight implications for drug targeting https://bit.ly/37MMxZa

Cite this article as: Allen RJ, Guillen-Guio B, Croot E, *et al*. Genetic overlap between idiopathic pulmonary fibrosis and COVID-19. *Eur Respir J* 2022; 60: 2103132 [DOI: 10.1183/13993003.03132-2021].

This single-page version can be shared freely online.

Copyright ©The authors 2022. For reproduction rights and permissions contact permissions@ersnet.org

Received: 10 Dec 2021
Accepted: 7 April 2022

To the Editor:

Coronavirus disease 2019 (COVID-19) is an infectious disease potentially leading to long-lasting respiratory symptoms and has resulted in over 4 million deaths worldwide. Idiopathic pulmonary fibrosis (IPF) is a chronic interstitial lung disease (ILD) characterised by an aberrant response to alveolar injury leading to progressive scarring of the lungs. Individuals with ILD are at a higher risk of death from COVID-19 [1].
